# Physical modeling of nucleosome clustering in euchromatin resulting from interactions between epigenetic reader proteins

**DOI:** 10.1073/pnas.2317911121

**Published:** 2024-06-20

**Authors:** Joseph G. Wakim, Andrew J. Spakowitz

**Affiliations:** ^a^Department of Chemical Engineering, Stanford University, Stanford, CA 94305; ^b^Department of Materials Science and Engineering, Stanford University, Stanford, CA 94305; ^c^Biophysics Program, Stanford University, Stanford, CA 94305; ^d^Department of Applied Physics, Stanford University, Stanford, CA 94305

**Keywords:** chromatin, epigenetics, euchromatin organization, polymer physics

## Abstract

Euchromatin is an accessible phase of genetic material containing genes that encode proteins with increased expression levels. The structure of euchromatin in vitro has been described as a 30-nm fiber formed from ordered nucleosome arrays. However, recent advances in microscopy have revealed an in vivo euchromatin architecture that is much more disordered, characterized by variable-length linker DNA and sporadic nucleosome clusters. In this work, we develop a theoretical model to elucidate factors contributing to the disordered in vivo architecture of euchromatin. We begin by developing a 1D model of nucleosome positioning that captures the interactions between bound epigenetic reader proteins to predict the distribution of DNA linker lengths between adjacent nucleosomes. We then use the predicted linker lengths to construct 3D chromatin configurations consistent with the physical properties of DNA within the nucleosome array, and we evaluate the distribution of nucleosome cluster sizes in those configurations. Our model reproduces experimental cluster-size distributions, which are dramatically influenced by the local pattern of epigenetic marks and the concentration of reader proteins. Based on our model, we attribute the disordered arrangement of euchromatin to the heterogeneous binding of reader proteins and subsequent short-range interactions between bound reader proteins on adjacent nucleosomes. By replicating experimental results with our physics-based model, we propose a mechanism for euchromatin organization in the nucleus that impacts gene regulation and the maintenance of epigenetic marks.

## Significance

Control over the spatial organization of euchromatin is essential for the determination and maintenance of cell identity across eukaryotic organisms. Despite important roles in cell-fate determination, the mechanisms governing euchromatin organization remain unclear. In this work, we develop a polymer physics model that recapitulates the heterogeneous architecture of euchromatin observed in vivo. Our results suggest that sparse patterns of repressive histone marks, preferential binding of reader proteins to those marks, and local interactions between bound reader proteins cause euchromatin to adopt its clustered arrangement. The mechanistic understanding generated by our research provides fundamental insight into the architecture of chromatin in living cells that is responsible for controlling protein expression and establishing cell identity.

There exist about 200 cell types in the human body with distinct physical and functional features, and transcriptomic subtyping reveals far more cellular diversity (1). Though somatic cells vary in size, shape, and function, they all share the same genetic code within an individual organism. Differences in cell identity are not caused by variations in DNA sequence but rather by differences in gene expression levels. Controlled organization of DNA in a dense nucleoplasm enables physical regulation of gene expression, driving cell differentiation and dictating cell identity (2).

With ∼3.2 billion base pairs (bp), the DNA in a human somatic cell would extend approximately two meters if stretched linearly (3). This genetic material is compacted into a nucleus that is typically ∼10 microns in diameter (4, 5). To condense the genetic material, DNA wraps around histone octamers to form nucleosomes, and chains of nucleosomes arrange into fibers called chromatin. Further condensation of chromatin is governed by regulatory mechanisms collectively known as epigenetics, in which chemical modifications to DNA and histones (called epigenetic marks) are preferentially bound by epigenetic “reader” proteins (6).

Trimethylation of the lysine located at the ninth residue on histone H3 (H3K9me3) is a predominant, repressive histone mark associated with chromatin condensation. H3K9me3 marks are recognized and preferentially bound by the chromodomain at the N-terminus of heterochromatin protein 1 (HP1). Once bound to histone tails, HP1 proteins in spatial proximity oligomerize due to favorable interactions between their C-terminus chromoshadow domains (7–10). Differences in H3K9me3 mark abundance and associated HP1 binding induce chromatin to phase separate into loosely packed euchromatin and dense heterochromatin. Genes located in euchromatin are more easily accessible to transcription factors, promoting their expression, while genes in heterochromatin are inaccessible, leading to their suppression. Aberrations in gene regulation arising from chromosomal organization can cause cells to lose their identities, contributing to conditions such as aging, Alzheimer’s disease, obesity, and cancer (11–20).

Prevailing representations of chromatin observed in vitro depict a “30-nm fiber,” with nucleosomes forming regularly ordered arrays (21–25). The ordered arrangement of nucleosome arrays in eukaryotes is also suggested by in vivo X-ray diffraction studies, which report highly conserved diffraction peaks around 11.0, 6.0, 3.8, 2.7, and 2.1 nm in both interphase and metaphase chromatin (26, 27).

However, recent studies suggest that in vivo chromatin may exhibit a more heterogeneous architecture (28–30). Euchromatin appears to adopt an open configuration, characterized by disordered nucleosomes separated by variable lengths of linker DNA (29, 30). Sporadic nucleosome clusters, typically 5 to 24 nm in diameter, form throughout the euchromatic phase (30). The disordered, open architecture of euchromatin is distinct from the ordered compaction of nucleosomes in heterochromatin determined from previous studies (31). Dinucleosomes are reported to produce nucleosome arrays with “asymmetric 3D zig-zag” arrangements that contribute to the overall heterogeneity in chromatin structure (32–34). The long-range asymmetry along the chromatin fiber is a result of variability in DNA linker lengths and wrapping around the nucleosomes (33). In this work, we explore the physical mechanisms governing euchromatin architecture, and we identify factors that play a critical role in gene regulation.

H3K9me3 marks are deposited by histone methyltransferases (also called “epigenetic writers”), such as SUV39H1. As HP1 readers preferentially bind H3K9me3-rich domains, their chromoshadow domains attract epigenetic writers, which deposit additional H3K9me3 marks on nearby nucleosomes (35–40). As a result, heavily marked regions tend to be further methylated, and patterns of H3K9me3 marks are correlated along the chromatin fiber. Nucleosome sliding is proposed to modulate the density of H3K9me3 marks, affecting mark inheritance and spreading (41, 42). We propose that the heterogeneous arrangement of euchromatin arises from correlated patterns of H3K9me3 marks and local HP1 interactions that drive heterogeneous nucleosome positioning along the chromatin fiber.

**Fig. 1.**
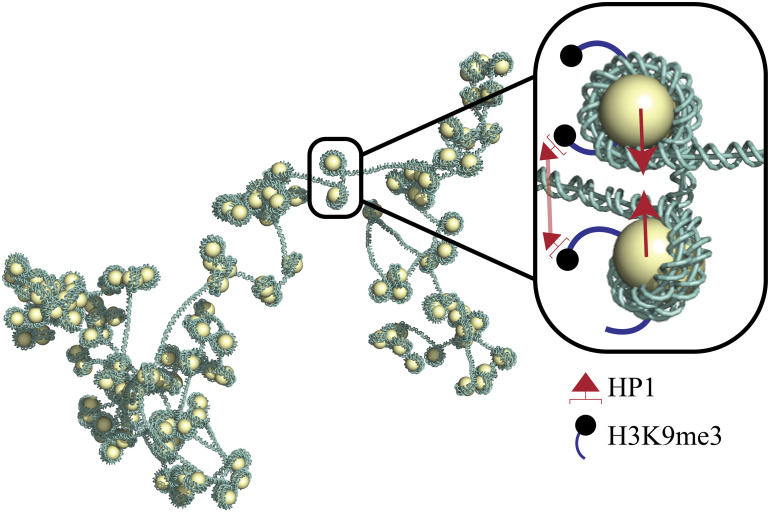
In our model, nucleosomes marked with H3K9me3 are preferentially bound by HP1 readers. Bound HP1s in close proximity oligomerize, driven by energetically favorable interactions. Nucleosomes are capable of sliding along the chromatin fiber. To accommodate favorable interactions, adjacent nucleosomes bound by HP1 tend to slide into proximity with each other. As a result, clusters of nucleosomes tend to form around regions rich in H3K9me3 marks, which are more preferentially bound by HP1.

We develop a model of nucleosome positioning based on heterogeneous, experimentally determined patterns of H3K9me3 marks and associated HP1 interactions in euchromatin. We then perform Monte Carlo (MC) simulations to sample patterns of linker lengths based on thermodynamic driving forces associated with our HP1-binding model. With these linker lengths, we construct ensembles of 3D euchromatin configurations that are governed by the physical properties of bare DNA and the geometry of DNA wrapped around histones. The intrinsic twist of DNA and kinks incurred by the DNA entry/exit angle at each nucleosome directly influence the geometry of the chromatin fiber (43). Fig. 1 provides a schematic of our model, which captures multiple physical effects and geometric factors governing euchromatin organization. We extract nucleosome cluster-size distributions from our ensembles of 3D chromatin configurations and show that the euchromatin configurations predicted by our model match experimental cluster-size distributions measured by ChromEMT (30). Our results suggest that heterogeneous epigenetic mark patterns and local reader-protein interactions are sufficient to explain the clustered arrangement of nucleosomes within euchromatin.

## Methods

## Materials and Methods

We develop a physical model to predict nucleosome organization in euchromatin, accounting for the heterogeneous binding of HP1 that drives local nucleosomal clustering. Our approach can be described in six steps. We first 1) initialize a 1D nucleosome array with a fixed pattern of H3K9me3 marks and 2) define a thermodynamic model of cooperative HP1 binding, accounting for heterogeneity in epigenetic patterning and local HP1 interactions. We then 3) develop an MC simulation to sample DNA linker lengths within the nucleosome array based on thermodynamic principles. 4) Using our predicted sequences of linker lengths, we initialize 3D chromatin configurations based on the kinked twistable wormlike chain (tWLC) model. The kinked tWLC model captures the physical behavior of linker DNA and the geometric contribution of the histone octamer (44). We next 5) use MC simulation to incorporate long-range steric interactions within nucleosome clusters. Finally, we 6) identify nucleosome clusters in our 3D chromatin configurations, evaluate their diameters, and compare the results to experimental data (30).

### Initialize Nucleosome Array.

We model euchromatin architecture arising from the binding of HP1 to H3K9me3. H3K9me3 is a well-established, repressive histone mark located in the tail region of the H3 core histone protein. Although H3K9 can be unmarked, mono-, di-, or tri-methylated, we simplify our model to only consider unmarked and tri-methylated H3K9, which captures the prevailing physical effects of heterogeneous HP1 binding and nucleosome clustering. We initialize a 1D chain of nucleosomes patterned with H3K9me3 marks. The identity of each nucleosome i in our model is given by $s_{i}\in \left\{0,1,2\right\}$, indicating whether zero, one, or two of its histone tails are modified with the mark. Based on our analyses of experimental chromatin immunoprecipitation sequencing data (45–47), the correlation in H3K9me3 marks along the chromatin fiber is approximated by an exponential function (see *SI Appendix*, Fig. S3 for demonstration). We vary the fraction of histone tails modified by H3K9me3 to reflect genomic heterogeneity in mark abundance (see *SI Appendix*, Fig. S2 for an example) (48–51).

### Model Cooperative HP1 Binding.

The binding of HP1 to marked and unmarked nucleosomes has been experimentally characterized by Canzio et al. (8), providing a quantitative determination of the thermodynamic properties for HP1 binding energy and cooperativity. HP1 reader proteins preferentially bind to H3K9me3 marks with an energetic preference of $\epsilon_{\text{m}}=-1.5k_{B}T$. Cooperativity in binding multiple HP1 readers in close proximity is captured by a favorable interaction energy $J_{\text{int}}=-3.92k_{B}T$, which applies both when HP1 readers are bound to the same nucleosome and nucleosomes in close spatial proximity (discussed further below).

In this work, we model HP1 binding using a grand-canonical ensemble, where unbound HP1s in the nucleoplasm are represented as a reservoir with fixed chemical potential. The chemical potential is given by $\mu_{\text{HP1}}\;=\;k_{B}T\log\left({\left[\text{HP1}\right]}_{\text{free}}\right)$, where ${\left[\text{HP1}\right]}_{\text{free}}$ is the concentration of unbound (i.e., free) HP1 in the nucleoplasm. Each nucleosome has two histone tails, and each histone tail may be bound by one HP1 reader. For the ith nucleosome, the HP1 binding state $\sigma_{i}\in \left\{0,1,2\right\}$ indicates the number of tails that are bound by HP1. We define the binding free energy $\phi_{\text{bind}}\left(\sigma_{i}\right)$ for a given number of marked tails $s_{i}$ and number of HP1s bound $\sigma_{i}$, which captures the free energy of HP1 binding to a single nucleosome and is determined using statistical mechanics arguments to be

[1] $$\begin{array}{c} \beta \phi_{\text{bind}}\left(\sigma_{i}\right)=\left\{\begin{array}{cc} 0 & \sigma_{i}=0,\text{all}\;s_{i},\\ -\beta \mu_{\text{HP1}}-\log2 & \sigma_{i}=1,s_{i}=0,\\ -\beta \mu_{\text{HP1}}-\log\left[1+\exp\left(-\beta \epsilon_{\text{m}}\right)\right] & \sigma_{i}=1,s_{i}=1,\\ -\beta \mu_{\text{HP1}}+\beta \epsilon_{\text{m}}-\log2 & \sigma_{i}=1,s_{i}=2,\\ -2\beta \mu_{\text{HP1}}+s_{i}\epsilon_{\text{m}}+J_{\text{int}} & \sigma_{i}=2,\text{all}\;s_{i}, \end{array}\right. \end{array}$$

where $\beta =1/(k_{B}T)$.

Each pair of HP1 readers that are bound to adjacent nucleosomes in close spatial proximity experience a favorable interaction of $J_{\text{int}}=-3.92k_{B}T$. We assume that bound HP1s only interact between adjacent nucleosomes if those nucleosomes are separated by a linker DNA length $l\leq l_{c}$. The cutoff length $l_{c}$ is set to 15 bp to approximate the range for which HP1 bridging can occur between neighboring nucleosomes without inducing considerable deformation of linker DNA (32). The binary variable $\gamma_{i}\in \left\{0,1\right\}$ indicates whether the linker length $l_{i}$ between the i and i+1 nucleosomes is within the 15-bp cutoff ($\gamma_{i}=1$ for $l_{i}\leq l_{c}$) or outside the 15-bp cutoff ($\gamma_{i}=0$ for $l_{i}>l_{c}$). The inter-nucleosomal interaction free energy $\phi_{\text{int}}\left(\sigma_{i},\sigma_{i+1}\right)$ is given by the number of HP1 readers bound to nucleosomes i and i+1 according to

[2] $$\begin{array}{c} \beta \phi_{\text{int}}\left(\sigma_{i},\sigma_{i+1}\right)=\beta J\gamma_{i}\sigma_{i}\sigma_{i+1}. \end{array}$$

The total binding free energy Φ=Φ({s},{l}) for a given epigenetic pattern $\left\{s\right\}=\left\{s_{1},s_{2},\ldots ,s_{M}\right\}$ and linker lengths $\left\{l\right\}=\left\{l_{1},l_{2},\ldots ,l_{M}\right\}$ is determined from the grand-canonical partition function

[3] $$\begin{array}{c} \Xi =\sum_{\sigma_{1}=0}^{2}\sum_{\sigma_{2}=0}^{2}\ldots \sum_{\sigma_{M}=0}^{2}\exp\left[-\sum_{i=1}^{M}\left(\beta \phi_{\text{bind}}+\beta \phi_{\text{int}}\right)\right]. \end{array}$$

We define the transfer function between nucleosomes i and i+1 to be

[4] $$\begin{array}{cc} T(\sigma_{i},\sigma_{i+1})= & \exp\left[-\frac{\beta \phi_{\text{bind}}\left(\sigma_{i}\right)}{2}-\frac{\beta \phi_{\text{bind}}\left(\sigma_{i+1}\right)}{2}\right.\\ & \left.-\beta \phi_{\text{int}}\left(\sigma_{i},\sigma_{i+1}\right)\right]. \end{array}$$

We evaluate the grand-canonical partition function Ξ by multiplying the matrices of M transfer functions, then taking the trace of the matrix product (i.e., using the transfer-matrix method). Thus, we adopt periodic boundary conditions, such that $\sigma_{M+1}=\sigma_{1}$, and we ensure that nucleosome arrays with M≥200 are sufficiently large to avoid finite-size effects. The total free energy is given by $\Phi =-k_{B}T\log\Xi$. Average HP1 binding $\left\langle \sigma_{i}\right\rangle$ at the ith nucleosome is also determined using the transfer-matrix method.

### Sample Linker Lengths.

The linker length $l_{i}$ gives the number of base pairs in the DNA connecting adjacent nucleosomes i and i+1 and can take integer values between one and infinity. Based on experimental observations (52, 53) and simulated results (54), we assume that the linker lengths follow an exponential distribution in the absence of interactions between adjacent nucleosomes. We specify an average linker length $l_{0}=$ 45 bp, which approximates the typical genome-wide linker length in eukaryotic cells (55–58). In the absence of interactions, the linker-length probability distribution scales as $P_{\text{link}}\left(l_{i}\right)∼\exp\left(-\lambda l_{i}\right)$, where $\lambda =-\log\left(1-1/l_{0}\right)$ and $l_{i}\in \left[1,\infty \right)$. To account for interactions, we introduce an additional Boltzmann-weighted probability based on the free energy change between the linker length being below the cutoff $l_{c}=$15 bp ($l_{i}\leq l_{c}$ and $\gamma_{i}=1$) or greater than the cutoff ($l_{i}>l_{c}$ and $\gamma_{i}=0$).

We develop an MC simulation to randomly select linker lengths based on our HP1-binding model and the base exponential linker-length distribution. For each step of the simulation, we select a random linker i along the chromatin fiber. We evaluate the free energy difference between configurations with $l_{i}\leq l_{c}$ and $l_{i}>l_{c}$ for that linker. For linker i, the free-energy difference ΔΦ is given by

[5] $$\begin{array}{c} \begin{array}{cc} \Delta \Phi =\; & \Phi (\left\{s\right\},\left\{\gamma_{1},\ldots ,\gamma_{i}=1,\ldots ,\gamma_{M}\right\})\\ - & \Phi (\left\{s\right\},\left\{\gamma_{1},\ldots ,\gamma_{i}=0,\ldots ,\gamma_{M}\right\}), \end{array} \end{array}$$

which modulates the linker-length distribution from its base exponential dependence. For a given free-energy difference, the linker-length probability distribution is given by

[6] $$\begin{array}{c} P_{\text{link}}\left(l_{i}\right)=\left\{\begin{array}{cc} \frac{1}{N}\exp\left(-\beta \Delta \Phi \right)\exp\left(-\lambda l_{i}\right) & l_{i}\leq l_{c},\\ \frac{1}{N}\exp\left(-\lambda l_{i}\right) & l_{i}>l_{c}, \end{array}\right. \end{array}$$

where

**Fig. 2.**
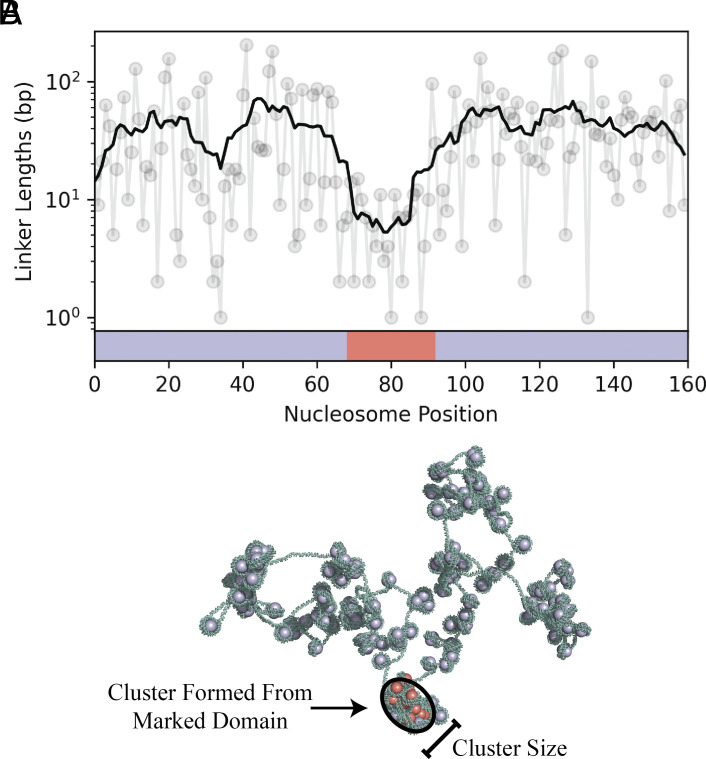
Heterogeneous patterns of H3K9me3 result in heterogeneous binding of HP1, causing clusters to form along the chromatin fiber. (*A*) To demonstrate our methods, we specify a pattern of H3K9me3 marks where 24 nucleosomes at the center of a nucleosome array are marked and the remaining nucleosomes are unmarked. This pattern is specified with the underlying colored bars, where marked and unmarked nucleosome positions are indicated in red and purple, respectively. We sample linker lengths by MC simulation and plot the resulting linker length at each position along the nucleosome array. The linker-length profile is smoothed using an 11-linker sliding window average. (*B*) We construct a 3D configuration based on the linker-length profile in (*A*). In the configuration, marked and unmarked nucleosomes are displayed in red and purple, respectively. Marked nucleosomes at the center of the nucleosome array are preferentially bound by HP1, and a cluster forms due to HP1 oligomerization. The cluster size is quantified by its minor principal axis.

[7] $$\begin{array}{c} N=\frac{z}{1-z}\left[\left(1-z^{l_{c}}\right)\exp\left(-\beta \Delta \Phi \right)+z^{l_{c}}\right], \end{array}$$

is a normalization constant to ensure $\sum_{l_{i}=1}^{\infty }P_{\text{link}}\left(l_{i}\right)=1$ and $z=\exp\left(-\lambda \right)$. We select $l_{i}$ from this governing distribution to complete a single Monte-Carlo step. We repeat the sampling process for randomly selected linkers until the linker-length distribution for the nucleosome array converges. Fig. 2*A* plots an example profile of methylation marks and resulting linker lengths for a 160-nucleosome section of the array.

### Initialize 3D Configuration.

Using thermodynamically determined linker lengths (discussed above), we initialize 3D chromatin configurations based on the kinked tWLC model (44). We do so by applying a chain-growth algorithm that randomly constructs the path of our chromatin fiber one base pair at a time. For each base pair of linker DNA, we randomly sample bending and twisting angles based on the elastic energy $E_{1}$, given by

[8] $$\begin{array}{c} \beta E_{1}=\frac{l_{p}}{2}\int_{0}^{L_{1}}ds\left(\omega_{1}^{2}+\omega_{2}^{2}\right)+\frac{l_{t}}{2}\int_{0}^{L_{1}}ds{\left(\omega_{3}-\tau \right)}^{2}, \end{array}$$

where $E_{1}$ is the energy for deforming a single base pair, $l_{p}=50$-nm is the persistence length of bare DNA, $L_{1}=0.332$-nm is the contour length per base pair of DNA, $l_{t}=100$-nm is the twist persistence length of bare DNA, and $\tau =2\pi {\left(10.5\;\text{bp}\right)}^{-1}$ is the natural twist of bare DNA (44). Bending and twisting angles are sampled from a Boltzmann-weighted probability distribution over all possible configurations, as discussed in *SI Appendix* of ref. 44. Each linker segment of DNA is “grown” by selecting these angles for each base pair, starting from the previous nucleosome and continuing to the next nucleosome. We assume that a constant 147-bp of DNA is wrapped around each nucleosome (59–61). This fixes the relative entry and exit positions and orientations of DNA around each nucleosome, introducing a kink in the 3D configuration each time a nucleosome is encountered. The process of growing linker DNA and introducing a kink at each nucleosome is repeated until the full nucleosome array is assembled (see Fig. 2*B* for an example configuration). The chain growth algorithm described above is discretized at the level of individual base pairs. We use this model to render detailed 3D configurations of the chromatin fiber. However, the algorithm does not account for steric interactions between nucleosomes, which can have meaningful effects on cluster size distributions (*SI Appendix*).

### MC Simulation of 3D Nucleosome Arrays.

We then develop an MC simulator for the 3D organization of the nucleosome array. Our MC simulations are discretized at nucleosome resolution. The positions and orientations of each nucleosome are initialized using the chain growth algorithm described above. During the simulation, we iteratively apply geometric transformations to random segments of nucleosomes. After each transformation, we evaluate the free-energy change of each affected linker DNA segment using the discrete stretchable, shearable, twistable wormlike chain model (62, 63). We assume that the geometry of DNA wrapped around each nucleosome is fixed, and we evaluate the elastic energy of each linker from the exiting position of one nucleosome to the entering position of the next. We include an energy associated with steric interactions using the Lennard-Jones repulsive potential below:

[9] $$\begin{array}{c} V=\sum_{i}^{N-1}\sum_{j=i+1}^{N}\left\{\begin{array}{cc} V_{0}\left[{\left(\frac{2R}{D_{\mathrm{ij}}}\right)}^{12}-2{\left(\frac{2R}{D_{\mathrm{ij}}}\right)}^{6}+1\right] & D_{\mathrm{ij}}<2R,\\ 0 & D_{\mathrm{ij}}\geq 2R, \end{array}\right. \end{array}$$

where V is the total repulsive potential due to overlapping nucleosomes, N is the number of nucleosomes in the array, $D_{\mathrm{ij}}=\left|\right|{\vec{r}}_{i}-{\vec{r}}_{j}\left|\right|$ is the distance between nucleosomes i and j, R=4.19 nm is the radius of a nucleosome (44), and $V_{0}=1.0\;k_{B}T$ is a scaling of the repulsive potential. We sample 3D chromatin configurations based on the free energy contributions from polymer elasticity and nucleosome sterics to evaluate cluster size distributions (see *SI Appendix* for more details). In *Results*, we show that our model recapitulates cluster size distributions observed in experiments, supporting the validity of our approach.

### Characterize Nucleosome Clusters.

We define clusters in a chromatin configuration as continuous sequences of four or more nucleosomes, each separated by up to $l_{c}=$15-bp of linker DNA. To evaluate the size of each cluster i, we begin by projecting the cluster onto a 2D plane (XY, XZ, or YZ) and computing the 2D inertia matrix I using the equation:

[10] $$\begin{array}{c} I=\sum_{j=1}^{N_{i}}\left({\vec{x}}_{j}-{\vec{x}}_{c}^{\left(i\right)}\right)⊗\left({\vec{x}}_{j}-{\vec{x}}_{c}^{\left(i\right)}\right), \end{array}$$

where $N_{i}$ is the number of nucleosomes in the cluster, ${\vec{x}}_{c}^{\left(i\right)}\in R^{2}$ is the center of mass of the cluster, and ${\vec{x}}_{j}\in R^{2}$ is the position of nucleosome j in the cluster. We then take the square root of the smallest eigenvalue of I. This represents the minor principal axis of the nucleosomes in the cluster and is akin to the experimental measurement of chromatin widths on 2D tomography slices. For a given sequence of linker lengths, we generate an ensemble of 3D chromatin configurations, project the configurations onto XY, XZ, and YZ planes, and evaluate the cluster size distribution for the ensemble from the projections. We investigate how the cluster size distribution varies with H3K9me3 abundance and HP1 chemical potential.

## Results

In this work, we show that cooperative HP1 binding can lead to the localized condensation of marked nucleosomes in euchromatin. As a result, euchromatin exhibits a heterogeneous architecture characterized by sporadic nucleosome clusters (30), which differs from the conventional 30-nm fiber model (22). We develop a physical model of cooperative HP1 binding to evaluate potential mechanisms driving nucleosome clustering. By fixing the chemical potential of HP1 and varying the concentration of H3K9me3 marks, we reproduce experimentally observed cluster-size distributions. These conditions reflect an environment with a shared pool of unbound reader proteins and spatial heterogeneity in epigenetic patterning, consistent with what is observed in eukaryotic nuclei. By reproducing experimental nucleosome cluster-size distributions with biologically relevant parameters, our physical model captures mechanisms governing euchromatin organization.

### H3K9me3 Abundance.

**Fig. 3.**
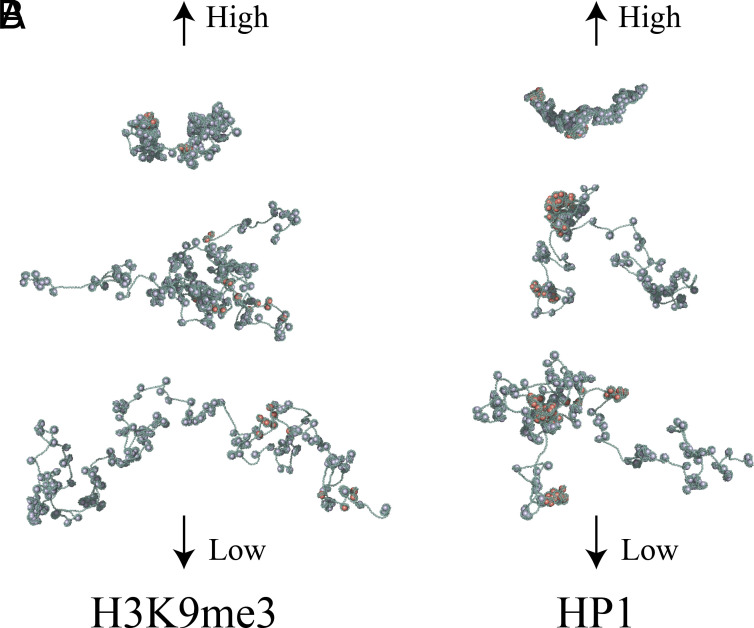
Euchromatin clusters depend on H3K9me3 mark abundance and HP1 concentration. (*A*) The fraction of histone tails marked with H3K9me3 varies from low to high values (0 to 2%, 8 to 10%, and 14 to 16%, from *Bottom* to *Top*). We keep the HP1 chemical potential (i.e., concentration) constant at a value of −9.7$k_{B}T$. We find that increasing the abundance of H3K9me3 marks induces cluster formation. (*B*) We then vary the concentration of HP1 from low to high values (chemical potentials of −9.8$k_{B}T$, −9.7$k_{B}T$, and −9.6$k_{B}T$, from *Bottom* to *Top*). We keep the fraction of histone tails marked with H3K9me3 fixed to values between 10 and 12%. As the concentration of HP1 increases, the nucleosome clusters tend to increase in size, even with a consistent abundance of H3K9me3 marks.

Spatial heterogeneity in chromosomal organization driven by epigenetic patterning is a hallmark of eukaryotic nuclei. In most eukaryotic cells, heterochromatin is concentrated towards the nuclear periphery, and nucleosome densities tend to increase radially outward. Notably, heterochromatin contains genomic regions that are rich in multiple repressive epigenetic marks, including H3K9me3. We capture spatial heterogeneity in H3K9me3 marks in our model of euchromatin. After characterizing concentrations of H3K9me3 in euchromatic domains (*SI Appendix*), we simulated euchromatin strands where 0 to 2%, 8 to 10%, and 14 to 16% of histone marks are marked, varying from “low” to “high” levels of H3K9me3 in euchromatin. We fix the chemical potential of HP1, representing a shared pool of reader proteins available for binding. Between the low and high levels of H3K9me3, nucleosomes collapse into dense clusters that tend to align with the marked domains. Fig. 3*A* plots configurations for 160-nucleosome segments from simulations of 1,000-nucleosome arrays with low to high levels of H3K9me3.

### HP1 Chemical Potential.

We assume that a pool of unbound (“free”) HP1 is distributed throughout the nucleoplasm, producing a chemical potential that modulates HP1 binding. A greater concentration of free HP1 results in a greater chemical potential and a higher propensity to bind the chromatin fiber. To evaluate the role of HP1 chemical potential on euchromatin organization, we simulate chromatin strands with HP1 chemical potentials of −9.8$k_{B}T$, −9.7$k_{B}T$, and −9.6$k_{B}T$. These conditions promote “low,” “moderate,” and “high” levels of HP1 binding to the chromatin fiber, respectively, and capture the transition from loose to condensed euchromatin. For these simulations, we mark 10 to 12% of histone tails with H3K9me3, consistent with observed H3K9me3 concentrations in euchromatin (*SI Appendix*). At a chemical potential of −9.8$k_{B}T$, HP1 binding aligns with patterns of H3K9me3 marks, and marked domains tend to form clusters while unmarked domains do not. Meanwhile, at an HP1 chemical potential of −9.6$k_{B}T$, HP1 binds unmarked nucleosomes near the boundary of a marked domain. As a result, nearby marked domains are bridged together, forming a shared cluster of nucleosomes (see Fig. 3*B* and *SI Appendix*).

### Euchromatin Clustering.

**Fig. 4.**
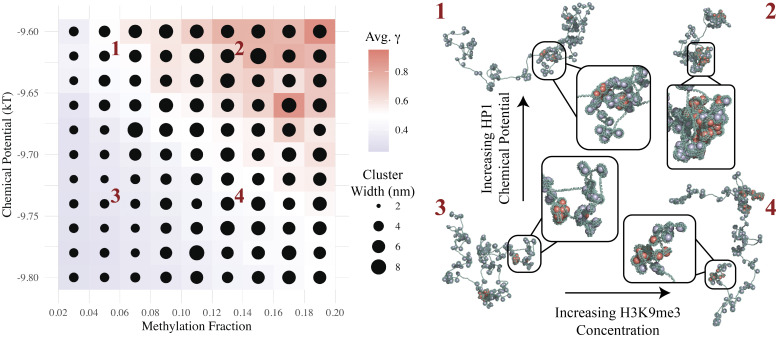
The abundance of H3K9me3 along the chromatin fiber and the concentration of HP1 in the nucleus affect the linker-length and cluster-size distributions. By tuning mark abundance and HP1 chemical potential, we capture the transition between loose and clustered architectures of euchromatin. In the plot, the color indicates the average fraction of linkers ≤15-bp in length (allowing HP1-HP1 interactions) from ensembles of chromatin fibers that are 1,000 nucleosomes long. The linker lengths are collected from our 1D model of nucleosome positioning. The size of each point reflects the average cluster size from ensembles of 3D chromatin fibers that are 200 nucleosomes long. Examples of 3D configurations are included for the four conditions labeled in the *Left* plot. In the configurations, marked and unmarked nucleosomes are shown in red and purple, respectively.

**Fig. 5.**
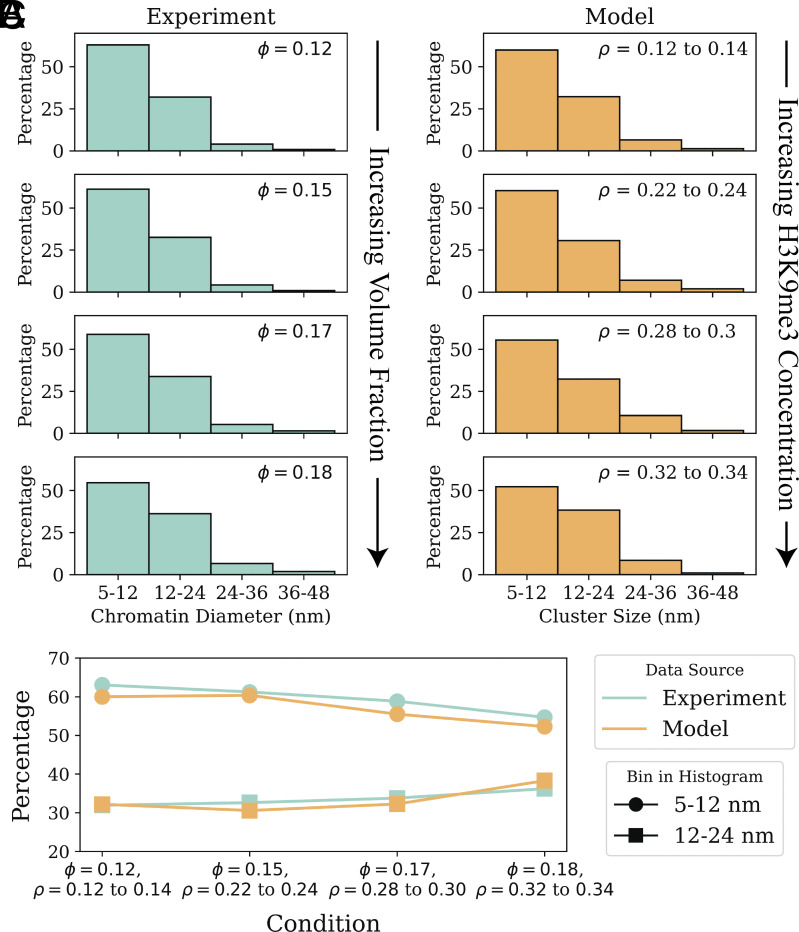
Our model reproduces experimental cluster-size distributions observed by Ou et al. with ChromEMT (30). (*A*) Electron micrograph tomography images collected by Ou et al. with ChromEMT are used to generate histograms of chromatin diameters in different voxels of the cell nucleus. We include histograms from ref. 30 for voxels with increasing volume fractions (ϕ), where H3K9me3 is thought to be at greater concentrations in most human cells. (*B*) We then plot similar distributions of chromatin cluster sizes obtained from our simulations of nucleosome positioning. The distributions represent different fractions of histone tails modified by H3K9me3 (ρ) and a fixed HP1 chemical potential of −9.66 $k_{B}T$. (*C*) We also plot the percentage values from the first two bins of each histogram to demonstrate a rightward shift in the cluster-size distribution associated with increased ϕ and ρ, as observed in both experiments and our model.

As demonstrated above, nucleosome clustering is affected by changes in both H3K9me3 concentration and HP1 chemical potential. We evaluate how combinations of both conditions affect euchromatin compaction. Fig. 4 plots the mean nucleosome cluster size and the fraction of DNA linker lengths ≤15-bp for various concentrations of H3K9me3 and chemical potentials of HP1. We capture a transition between loose and clustered euchromatin configurations that depends on both epigenetic patterning and reader protein abundance in the nucleoplasm. By fixing the HP1 chemical potential to −9.66 $k_{B}T$ and varying the concentration of H3K9me3, we approximately reproduce experimental cluster size distributions observed by Ou et al. (30). Fig. 5 plots cluster size distributions observed by Ou et al. next to those predicted by our model for four H3K9me3 concentrations. We discuss the biological significance of these findings in detail below.

## Discussion

Recent advances in microscopy have enabled the characterization of euchromatin architecture in vivo, revealing disordered chains of nucleosomes with sporadic clusters (28, 30, 64, 65). The heterogeneous arrangement of euchromatin differs from the conventional 30-nm chromatin fiber model, which was based on in vitro observations (22). We develop a physical model of cooperative HP1 binding that recapitulates the disordered architecture of euchromatin, thereby offering a potential mechanism for euchromatin organization.

We begin with a nucleosome array patterned with exponentially correlated H3K9me3 marks. We assume the timescale for mark conferral far exceeds the timescale for chromatin reorganization. Therefore, we fix the epigenetic pattern in each simulation. Marked nucleosomes are preferentially bound by HP1 readers, and bound HP1 readers tend to oligomerize when in close spatial proximity. Since long-range nucleosome contacts in euchromatin are infrequent, we limit HP1 interactions to neighboring nucleosomes. Our model captures the dominant mechanism by which HP1 interactions dictate nucleosome positioning in euchromatin. However, because HP1 interactions are limited to neighboring nucleosomes, the model does not generalize to heterochromatic domains, where additional HP1 interactions occur between genomically distant nucleosomes in condensed regions.

We assign a 15-bp linker-length cutoff on HP1 oligomerization between adjacent nucleosomes. This limit reflects the range of HP1 bridging between neighboring nucleosomes before considerable deformation is incurred by the linker DNA (32). The strict 15-bp linker-length cutoff on HP1 interactions allows us to analytically evaluate the free energy of a configuration based on linker lengths and epigenetic patterning. In the absence of interactions, we assume that linker lengths are exponentially distributed (52–54). The addition of interactions leads to separate distributions for lengths ≤15-bp and >15-bp.

Using thermodynamically determined DNA linker lengths, we construct 3D chromatin configurations according to the kinked tWLC model (44). In doing so, we assume that linker DNA behaves as a semiflexible polymer and that the entry and exit orientations of DNA from each nucleosome are fixed. To account for steric interactions between nucleosomes, we apply a Lennard-Jones repulsive potential and sample 3D chromatin configurations by MC simulation (see *SI Appendix* for details). While this simple approach to capturing long-range interactions may affect fine-grained features of the chromatin architecture, we focus on larger-scale organization associated with clustering.

Our model is intended to isolate the effect of cooperative HP1 binding on nucleosome clustering. Our MC simulation is based on the observation that nucleosomes, in the absence of highly positioning sequences, exhibit exponentially distributed linker lengths. This distribution is observed in vivo and arises from a variety of different contributions, including thermal fluctuations of the nucleosomes as well as the active remodelers that drive nucleosome repositioning. We assume that HP1 oligomerization will bias this distribution under conditions where HP1 binds to neighboring nucleosomes, and we capture this effect based on a Boltzmann-weighted bias.

We do not account for heterogeneity in DNA wrapping around each nucleosome due to histone H1, nor do we account for chromatin remodelers. Histone H1 binds DNA near the entry and exit sites along the nucleosome core particles and functions to stabilize the nucleosome and compact the chromatin fiber (66–69). Chromatin remodelers actively alter nucleosome positioning and may promote or prevent HP1 interactions (70–73). Further theoretical development is needed to address the interplay between HP1 binding and nucleosome remodelers, given the non-equilibrium nature of their actions. Our model can be further expanded by accounting for the role of electrostatic interactions in chromatin folding. Varying ionic conditions will affect both electrostatic interactions between DNA segments as well as local geometric features of the nucleosomes (33, 34, 74–76). However, addressing these effects would require the incorporation of electrostatic interactions within the chromosome as well as with surrounding biopolymers and ionic species within the nucleus. Using our minimal representation of chromatin, we efficiently obtain cluster-size distributions consistent with those observed by Ou et al. (30). Further refinements to the model will be valuable in elucidating other structural features and biological functions.

We identify the epigenetic mark abundance and reader-protein concentration as potential drivers of nucleosome clustering (Fig. 4). With a greater fraction of histone tails marked with H3K9me3, there are more favorable sites for HP1 binding. With a greater chemical potential of HP1, there are more readers available for binding. Both conditions result in a stronger enthalpic drive to bind HP1 to the chromatin fiber, resulting in greater compaction.

Towards the nuclear periphery, H3K9me3 marks are more abundant and nucleosome clusters tend to be larger (30, 77–79). We fix the chemical potential of HP1 and vary the abundance of H3K9me3 marks (as in Fig. 5) to represent a nucleus with a shared pool of reader proteins and spatial heterogeneity in epigenetic patterning. Our model combines three experimentally observed factors: 1) H3K9me3 marks are exponentially correlated in euchromatin, 2) H3K9me3 is preferentially bound by HP1, and 3) HP1 oligomerizes with other HP1s in spatial proximity. With just these ingredients, our model reproduces experimental nucleosome cluster-size distributions observed by Ou et al. (30). Since our physical model reproduces experimental observations using biologically relevant parameters, the model reveals a mechanism consistent with euchromatin organization.

We acknowledge that other epigenetic marks and reader proteins also contribute to cluster formation. For example, Polycomb Repressive Complex 1 interacts with H3K27me3 marks in a similar manner to HP1 and H3K9me3. However, our analyses of ChIP-seq data show an abundance of highly correlated H3K9me3 marks throughout the chromosome, whereas H3K27me3 marks tend to be more isolated. Given the abundance and correlation of H3K9me3 marks, we focus on this mark in this study of nucleosome clustering with our minimal representation of the chromatin fiber. The observation, based on our model, that trace levels of epigenetic marks and reader proteins in euchromatin can lead to cluster formation prompts future exploration of the topic, during which we can explore the roles of other epigenetic marks.

The mechanisms driving cluster formation in euchromatin and heterochromatin are distinct. Compared to euchromatin, heterochromatin is dense in nucleosomes and reader proteins, characterized by more frequent long-range contacts. As a result, heterochromatic nucleosomes are less reliant on interactions between adjacent nucleosomes to form HP1 interactions. Therefore, while more compact overall, heterochromatic nucleosomes tend to be separated by longer linkers (80–82). With no shortage of nearby readers available to form interactions, entropic pressures dominate the architecture of heterochromatin, and looping becomes an important factor in chromatin organization. Future development of our model will include the addition of long-range HP1 interactions to study the effect of nucleosome density on linker-length distribution.

We have previously shown that chromatin looping can cause H3K9me3 mark spreading from heterochromatic to euchromatic domains (83, 84). This “epigenetic drift” can contribute to age-related diseases like Alzheimer’s disease and cancer (11–17, 19, 20). However, the precise effect of epigenetic drift on gene accessibility in euchromatin remains understudied. By combining our models of chromatin phase separation (63, 85), epigenetic mark heritability (83, 84, 86, 87), and euchromatin clustering, we will investigate the stability of euchromatin architecture near epigenetic domain boundaries. This follow-up study will capture the interplay between chromatin structure and epigenetic patterning, reflecting the behavior of methyltransferases that bind marked domains and promote local mark spreading. We hypothesize that nucleosome clusters grow in response to the loop-mediated spread of H3K9me3 into euchromatin and that chromatin remodelers play an important role in modulating linker-length distributions near epigenetic boundaries.

## Summary

We develop a physical model that captures the disordered architecture of euchromatin observed in vivo. Our model reflects cooperative HP1 binding, such that readers preferentially bind nucleosomes with H3K9me3 marks. We sample DNA linker lengths between adjacent nucleosomes based on local HP1 interactions. With thermodynamically determined linker lengths, we generate 3D euchromatin configurations and show that the cluster-size distributions in our configurations are consistent with experimental observations. Our results suggest that nucleosome positioning and associated chromatin compaction are affected by both H3K9me3 patterning and HP1 chemical potential. HP1 oligomerization causes nucleosome clusters to form where H3K9me3 marks are locally concentrated on the euchromatic fiber.

The mechanism driving cluster formation in euchromatin is distinct from compartmentalization in heterochromatin, where condensation is strongly influenced by long-range chromatin looping. Unlike in heterochromatic domains where there is an abundance of epigenetic readers available to form interactions, HP1 readers bound to nucleosomes within euchromatin have limited neighbors with which to interact. Therefore, the positioning of euchromatic nucleosomes relative to their neighbors dictates whether HP1s can oligomerize, and greater concentrations of HP1 tend to reduce average linker lengths.

Euchromatin contains the genetic code for vital proteins that determine cell identity, and the precise expression of these proteins is essential for maintaining proper cell function. The organization of euchromatin plays a pivotal role in gene expression. Our work offers a mechanistic explanation of euchromatin organization, providing fundamental insight into the biological function of chromosomal DNA with important implications for human health.

## Supplementary Material

Appendix 01 (PDF)

## Data Availability

Our MC simulation software for sampling linker lengths based on cooperative HP1 binding is available at https://github.com/JosephWakim/NucleoArr (88). Using fixed linker lengths, we initialize 3D chromatin configurations based on the kinked tWLC model, with an associated chain-growth model available at https://github.com/ajspakow/wlcstat (89). Once a 3D nucleosome array is initialized, we reduce steric clashes using our MC simulator available at https://github.com/SpakowitzLab/chromo (90). All other data are included in the manuscript and/or *SI Appendix*.
