# Supplementary material for: Physical modeling of nucleosome clustering in euchromatin resulting from interactions between epigenetic reader proteins
Source: Proc Natl Acad Sci U S A. 2024 Jun 20;121(26):e2317911121. doi: 10.1073/pnas.2317911121 (PMC11214050; doi:10.1073/pnas.2317911121)
Supplement: Supplementary file 1 — Appendix 01 (PDF) [file pnas.2317911121.sapp.pdf]

Supporting Information for

Physical Modeling of Nucleosome Clustering in Euchromatin Resulting From Interactions Between Epigenetic Reader Proteins

Joseph G. Wakim and Andrew J. Spakowitz

Andrew J. Spakowitz
E-mail: ajspakow@stanford.edu

This PDF file includes:

Supporting text
Figs. S1 to S8
SI References

Supporting Information Text

Establishing Epigenetic Mark Patterns. We model chromatin patterned with a prominent, repressive histone mark: histone H3, lysine 9 trimethylation (H3K9me3). We sample exponentially correlated patterns of H3K9me3 marks, representing segments of euchromatin (as justified in the next section). For each H3K9me3 mark pattern, we specify the expected fraction of histone tails modified with the mark. Assuming that the timescale of epigenetic mark spreading far exceeds the timescale of chromatin reorganization, we fix the pattern of epigenetic marks for the duration of each simulation. Let $l_m \in \mathbb{R}^+$ represent the mean correlation length of the exponential distribution governing H3K9me3 patterns, and let $f_m \in [0, 1]$ represent the expected fraction of histone tails with H3K9me3 marks. The identity of each nucleosome i in our model is given by $s_i \in \{0, 1, 2\}$, indicating whether zero, one, or two of its histone tails are modified with the mark. We begin by selecting the H3K9me3 state of the first nucleosome based on the fraction of histone tails with the mark:

$$P(s_0 = 0) = (1 - f_m)^2 \quad [1]$$

$$P(s_0 = 1) = 2f_m(1 - f_m) \quad [2]$$

$$P(s_0 = 2) = f_m^2 \quad [3]$$

The H3K9me3 mark state at each subsequent site i depends on the mark state at site $(i - 1)$. If the nucleosome at site $(i - 1)$ is marked, then the conditional probability of having at least one mark at site i is given by Eq. 4:

$$P\big(s_i > 0 \mid s_{i-1} > 0\big) = \big(1 - f_m\big)e^{-1/l_m} + \big(f_m\big) \quad [4]$$

Likewise, if the nucleosome at site $i - 1$ is unmarked, then the conditional probability of having no mark at site i is given by Eq. 5:

$$P\big(s_i = 0 \mid s_{i-1} = 0\big) = \big(f_m\big)\ e^{-1/l_m} + \big(1 - f_m\big) \quad [5]$$

Using Eqs. 4 and 5, we can determine if the nucleosome at site i has at least one mark. If the nucleosome is marked, then Eqs. 6 and 7 represent the probabilities of the nucleosome having exactly one and two marks, respectively. With these conditional probabilities, we define a Markov process for building up patterns of H3K9me3 marks.

$$P\big(s_i = 1 \mid s_i > 0\big) = \frac{2\big(1 - f_m\big)}{\big(2 - f_m\big)} \quad [6]$$

$$P\big(s_i = 2 \mid s_i > 0\big) = 1 - P\big(s_i = 1 \mid s_i > 0\big) = \frac{f_m}{\big(2 - f_m\big)} \quad [7]$$

Modeling Euchromatin. We evaluate the size distribution and methylation patterns of euchromatic domains for each chromosome based on chromatin immunoprecipitation sequencing (ChIP-seq) data from the ENCODE database (1, 2, 3). We distinguish euchromatic domains based on methods described in Ref. (4). The ChIP-seq data used in our analysis comes from a human lymphoblastoid cell line (based on publicly available data at ENCODE file accession ENCFF683HCZ). The size distributions for euchromatic domains on all chromosomes are plotted in Fig. S1. In our study, we model segments of euchromatin up to 1000 nucleosomes long. This segment size falls within the range of domain sizes for euchromatin observed experimentally and avoids significant finite-size effects. The fractions of histone tails marked with H3K9me3 in euchromatic domains on each chromosome are plotted in Fig. S2. In our study, we vary the fraction of histone tails marked with H3K9me3 from 0-34%, which approximately reflects what is observed experimentally and captures the transition toward a heterochromatic regime. As demonstrated by Fig. S3, H3K9me3 marks approximately follow an exponential correlation in euchromatic domains. Accordingly, we generate exponentially correlated patterns of H3K9me3 marks to model segments of euchromatin in our study.

Monte Carlo Simulation of 3D Nucleosome Array. We use Monte Carlo (MC) simulations to evaluate long-range steric interactions between nucleosomes in 3D configurations predicted by our chain growth model. During each MC simulation, we iteratively apply 3D geometric transformations ("moves") to random segments of the chromatin fiber. These transformations include single-nucleosome rotations, translations, crank-shaft rotations, and end-pivots (see Fig. S5 and Ref. (4) for details). With each MC move, we evaluate the change in free energy of the configuration. The free energy of the system includes steric contributions and elastic energy. To determine steric contributions to the free energy, we calculate pairwise distances between nucleosomes along the chromatin fiber and evaluate a Lennard-Jones repulsive potential given by Eq. 9 in the main text. Fig. S6 includes two examples demonstrating that the Lennard-Jones repulsive potential is effective in reducing steric overlap between nucleosomes over the course of an MC simulation. We evaluate the elastic energy of a configuration based on the kinked stretchable, shearable, twistable wormlike chain model with the free-energy equation reproduced below (4, 5, 6):

$$\mathcal{E}_{\text{elas.}}(\{\vec{R}_i, \vec{u}_i\}) = \sum_{i=2}^{N} \left[\frac{\epsilon_b}{2\Delta} \left| \vec{u}_i - \vec{u}_{i-1} - \eta \vec{R}_i^{\perp} \right|^2 + \frac{\epsilon_{\parallel}}{2\Delta} \left(\vec{R}_i \cdot \vec{u}_{i-1} - \Delta\gamma \right)^2 + \frac{\epsilon_{\perp}}{2\Delta} \left| \vec{R}_i^{\perp} \right|^2 + \frac{\epsilon_{\text{t}}}{2\Delta} (\Omega_i - \tau)^2 \right] \quad [8]$$

where $\vec{r}_i$ and $\vec{u}_i$ are the position and tangent vectors of the polymer at monomer i, $\vec{R}_i = \vec{r}_i - \vec{r}_{i-1}$, $\vec{R}_i^{\perp} = \vec{R}_i - (\vec{R}_i \cdot \vec{u}_{i-1})\vec{u}_{i-1}$, Ω_i is the twist angle between nucleosomes $i-1$ and i, $\tau = 2\pi(10.5 \text{ bp})^{-1}$ is the natural twist of bare DNA, and N is the number of nucleosomes in the chromatin fiber. The elastic energy is scaled by parameters ϵ_b, $\epsilon_{\parallel}$, $\epsilon_{\perp}$, ϵ_{t}, γ, and η, which depend on Δ, the number of persistence lengths separating adjacent beads of the polymer. We assume that the geometry of DNA wrapped around the nucleosome is fixed (6); there is a fixed relationship between the entering and exiting DNA positions and orientations at each nucleosome. Therefore, for each linker l along the chromatin fiber, we evaluate the elastic energy from the exiting position and orientation of nucleosome l to the entering position and orientation of the adjacent nucleosome $l+1$. Fig. S7 demonstrates the validity of our MC simulator. By neglecting steric interactions, our simulator produces configurations with mean squared end-to-end distances that are consistent with those derived from our chain growth algorithm for the kinked twistable wormlike chain (6).

Effect of Steric Interactions on Cluster Size Distributions. We initialize 3D chromatin configurations using a chain growth algorithm for the kinked stretchable, shearable, twistable wormlike chain. However, the chain growth algorithm does not account for steric interactions between nucleosomes. Steric interactions cause rightward shifts in the cluster size distributions predicted by our model. As steric interactions are accounted for by MC simulation, nucleosomes tend to move apart from one another and cluster sizes tend to increase. Fig. S8 exemplifies the rightward shift in cluster size distribution observed when steric interactions are addressed by MC simulation.

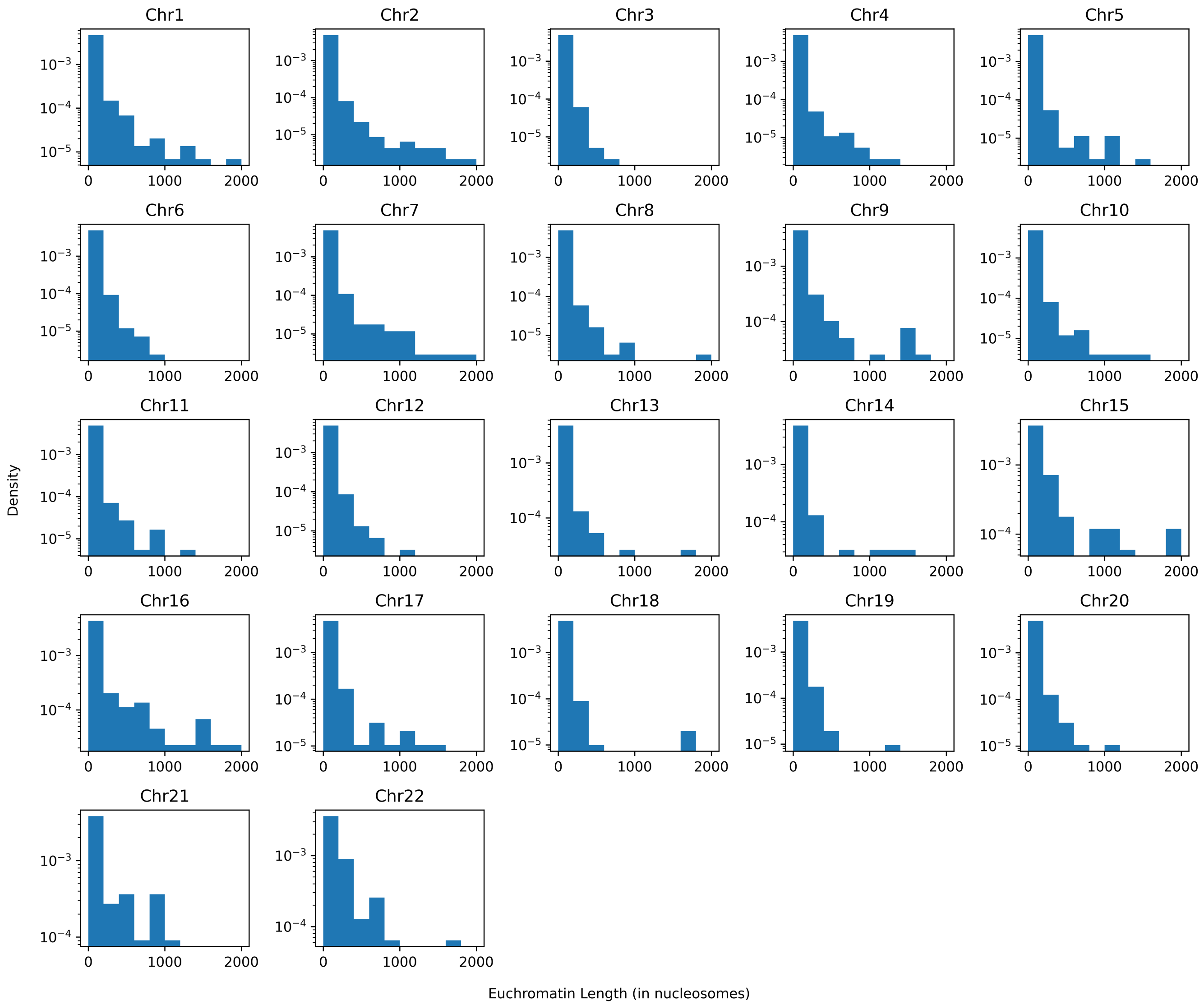

Fig. S1. We evaluate the size distributions of euchromatic domains across the genome. We classify euchromatic domains based on ChIP-seq data from the ENCODE database (1, 2, 3) using methods described in Ref. (4). The ChIP-seq data used in our analysis comes from a human lymphoblastoid cell line (ENCODE file accession ENCFF683HCZ). The histograms provide the number of nucleosomes in continuous euchromatic segments for each chromosome. To generate cluster size distributions, we simulate euchromatic domains that are 200 nucleosomes in length.

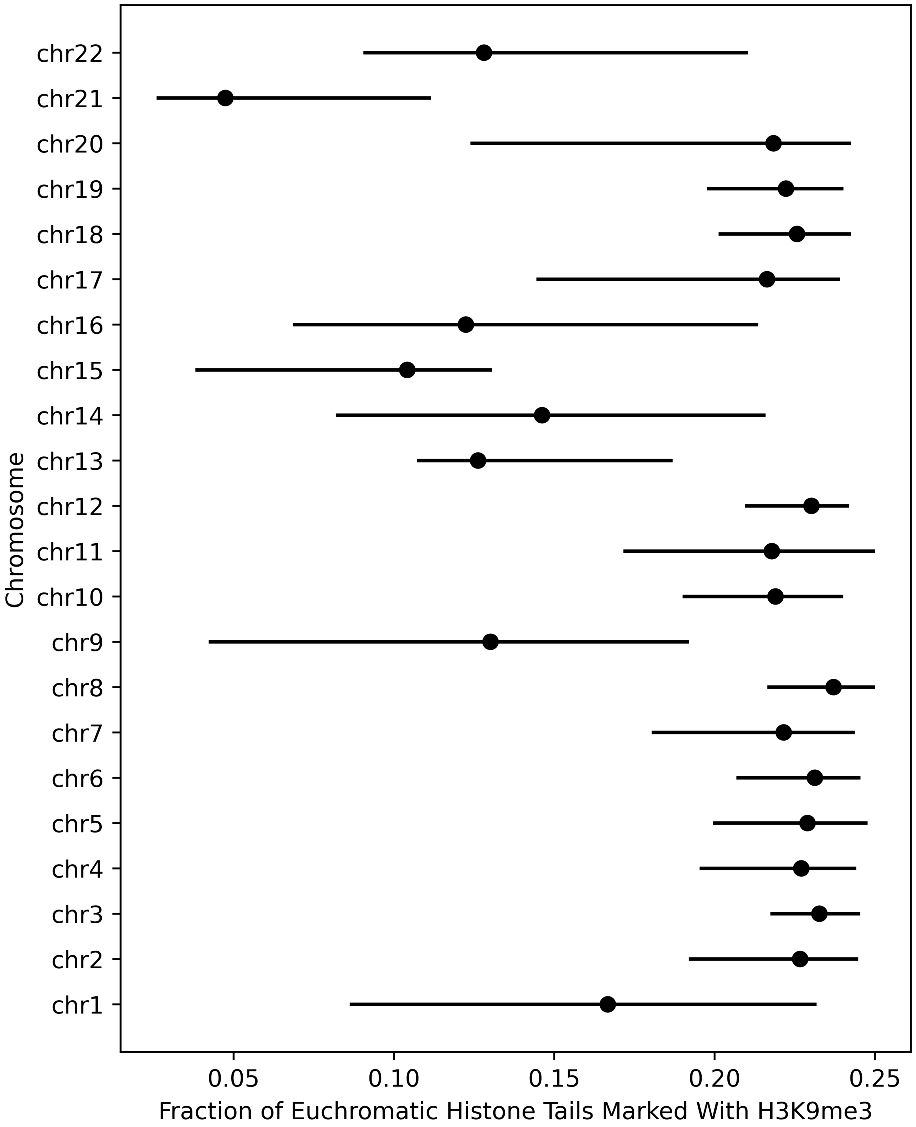

Fig. S2. We evaluate the fractions of histone tails that are marked with H3K9me3 in euchromatic domains across the genome. We classify euchromatic domains based on ChIP-seq data from the ENCODE database (1, 2, 3) using methods described in Ref. (4). The ChIP-seq data used in our analysis comes from a human lymphoblastoid cell line (ENCODE file accession ENCFF683HCZ). Among euchromatic domains that are at least 100 nucleosomes in length, we plot the median fraction of histone tails marked with H3K9me3 (as points) and the associated interquartile range (as error bars) by chromosome.

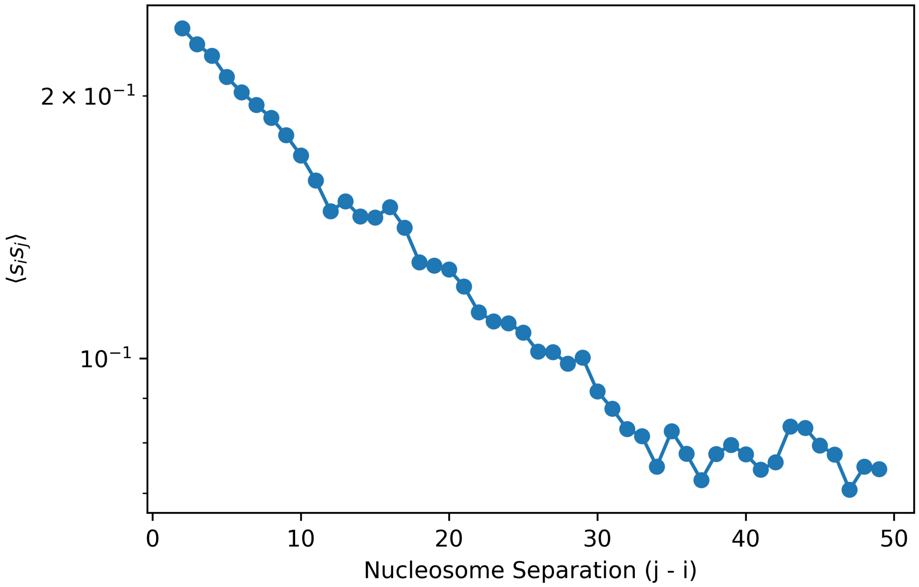

Fig. S3. We extract H3K9me3 mark patterns from euchromatic domains based on ChIP-seq data and evaluate the correlation between the marks. Among euchromatic domains that are at least 100 nucleosomes in length, H3K9me3 marks appear to be exponentially correlated along the chromatin fiber. Accordingly, we assume an exponential correlation between H3K9me3 marks when initializing euchromatic segments in our study.

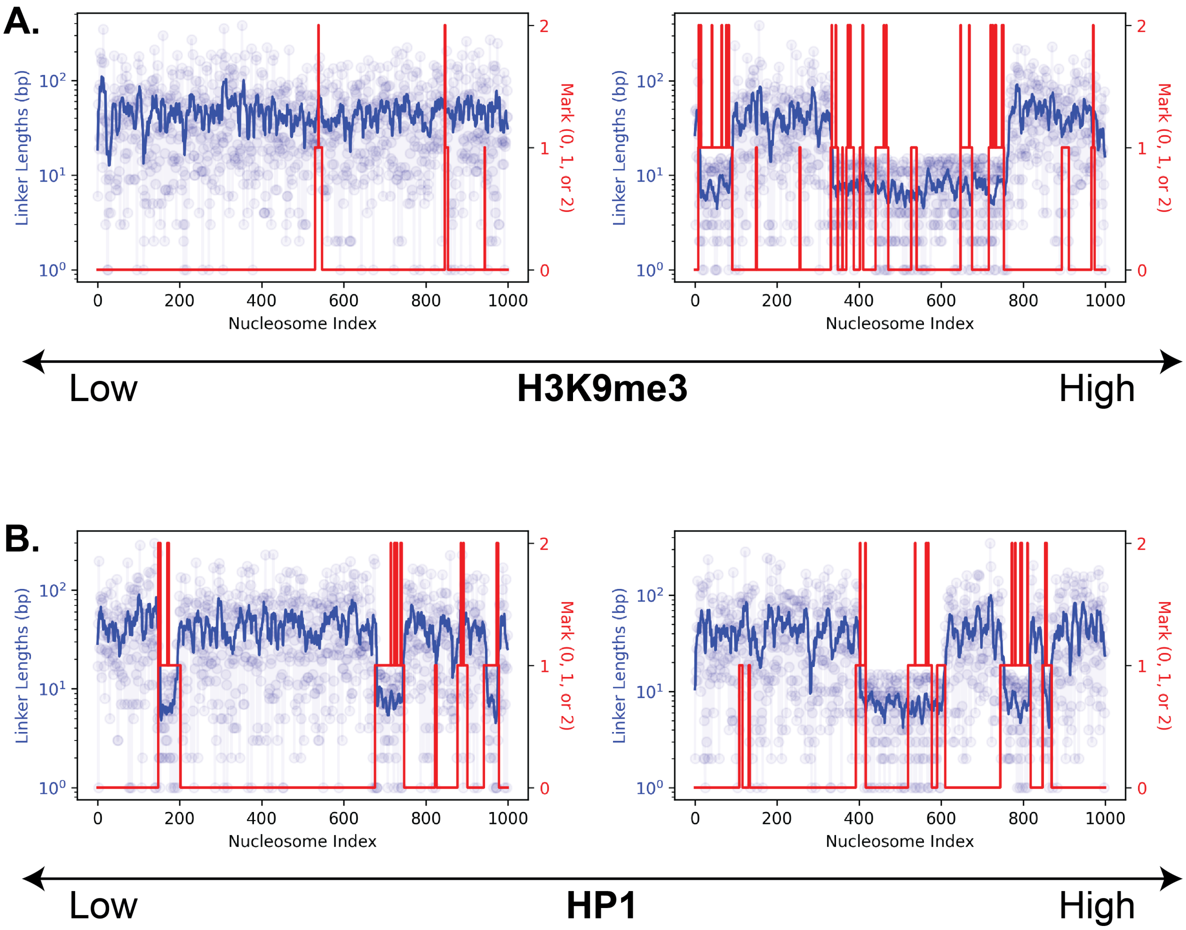

Fig. S4. Nucleosome clusters in euchromatin depend on H3K9me3 mark abundance and HP1 chemical potential. This figure includes profiles of H3K9me3 marks (in red) and DNA linker lengths (in blue) for 1000-nucleosome segments of euchromatin. The profiles represent realizations of equilibrated chromatin configurations under different sets of conditions. **(A)** We vary the fraction of histone tails marked with H3K9me3 between low and high values (0-2% and 14-16%, respectively). We keep the chemical potential of HP1 constant at a value of -9.7 kT. **(B)** We then vary the chemical potential of HP1 between low and high values (-9.8 kT and -9.6 kT, respectively). We keep the fraction of histone tails marked with H3K9me3 fixed to values between 10-12%.

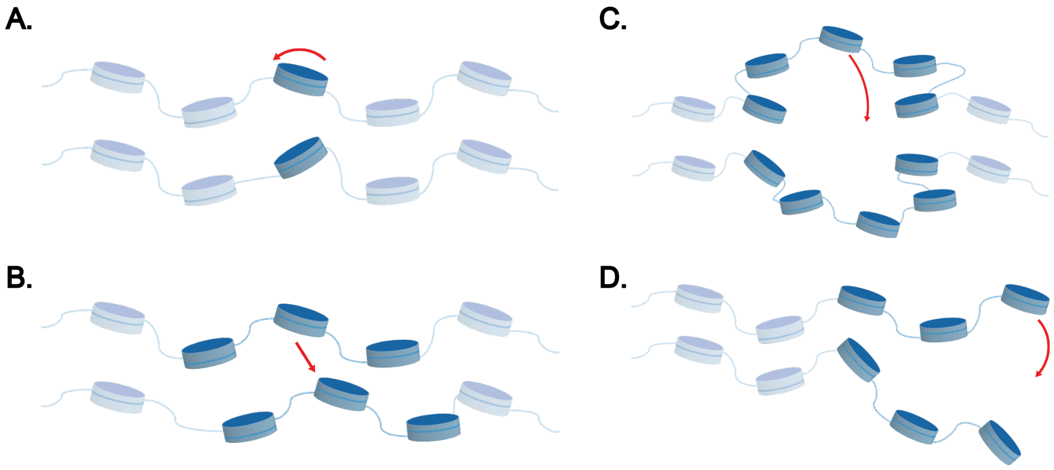

Fig. S5. Schematics of MC moves applied to reduce steric overlap in predicted chromatin configurations. **(A)** The single-bead rotation alters the orientation of a single nucleosome without affecting its position in space. **(B)** The translation ("slide") move alters the position of a nucleosome segment without affecting its orientation. **(C)** The crank-shaft move rotates a nucleosome segment about its end-to-end axis. **(D)** The end-pivot move rotates one of the two ends of the polymer about a random axis.

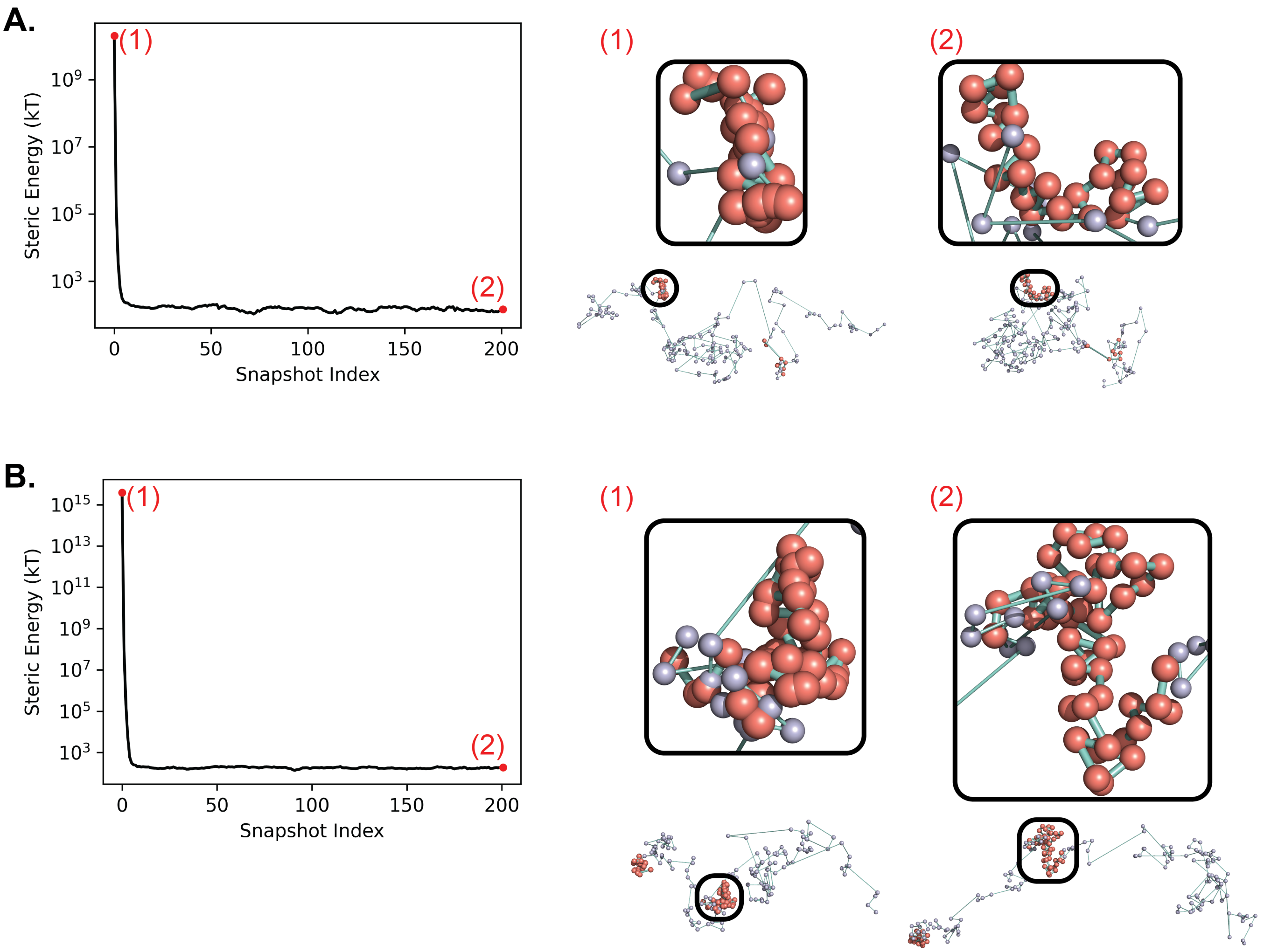

Fig. S6. We initialize MC simulations with chromatin configurations using a chain growth algorithm (6). While this chain growth algorithm obeys the bending and twisting probabilities of a twistable wormlike chain, the model does not take into account steric interactions between nucleosomes along the chromatin fiber. To reduce steric overlap between nucleosomes, we implement a Lennard-Jones repulsive potential (see Eq. 9 in main text). This potential drives the separation of overlapping nucleosomes. Here, we show the energy associated with steric interactions for two MC simulations of 200-nucleosome arrays. The simulations represent chromatin fibers with a -9.66 $k_B T$ HP1 chemical potential and **(A)** 8-10% and **(B)** 18-20% of histone tails marked with H3K9me3. We include snapshots of configurations at the start and end of each MC simulation to show the overall reduction in steric overlap. Beads in each snapshot indicate the positions of nucleosomes along the chromatin fiber. The figure demonstrates that the extent of steric overlap between nucleosomes was effectively reduced by MC simulation.

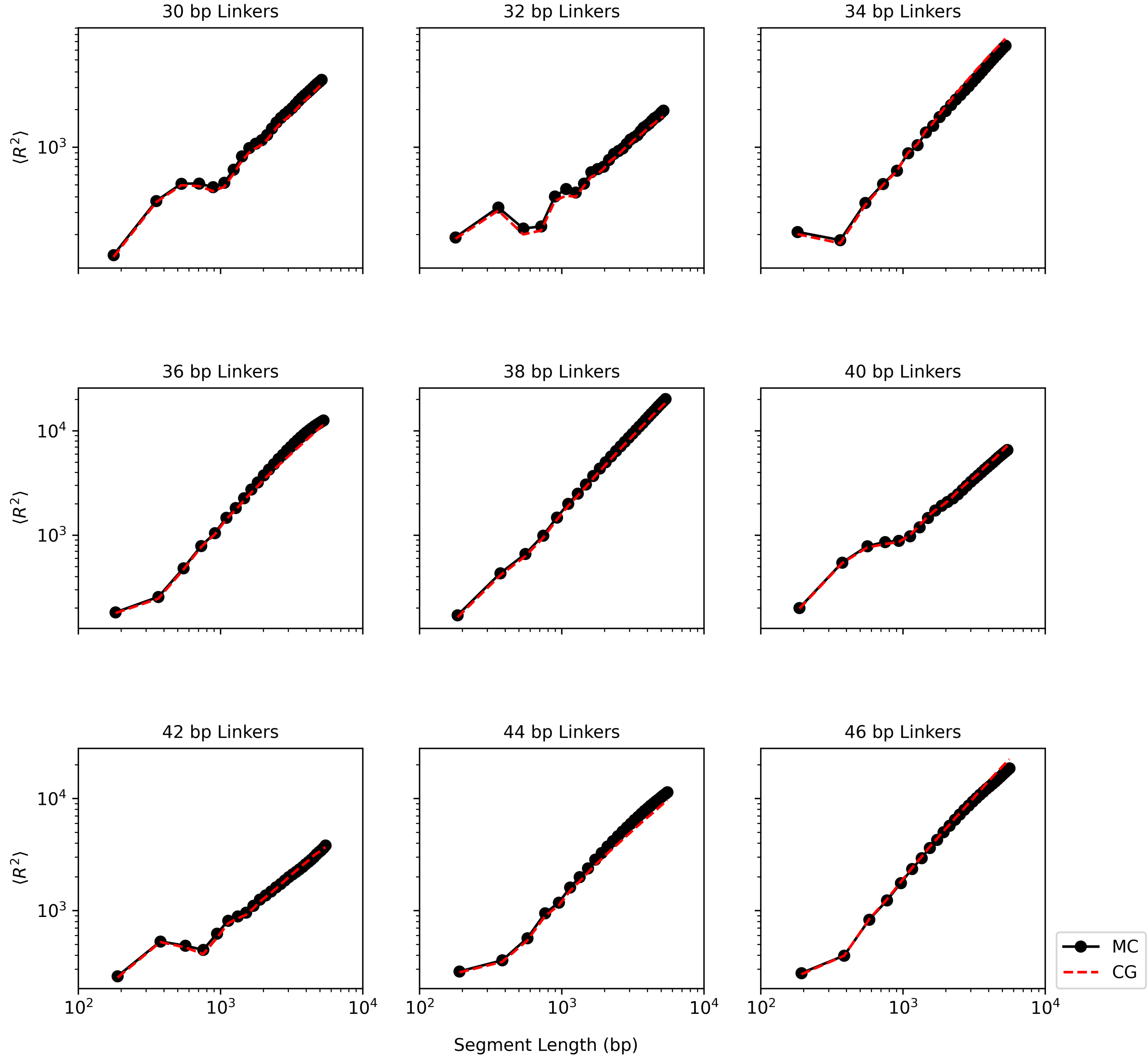

Fig. S7. To demonstrate the validity of our MC simulator for modeling the kinked stretchable, shearable, twistable wormlike chain, we simulate chromatin fibers with fixed linker lengths and compare the resulting mean squared end-to-end distances with those predicted by our chain growth algorithm (6). We model chromatin fibers with 50 nucleosomes. Our MC simulations are initialized as linear chains. For consistency with the chain growth algorithm, we neglect steric interactions in our MC simulations. Here, we demonstrate that the mean squared end-to-end distances obtained from our MC simulations ("MC") are consistent with those from our chain growth model ("CG").

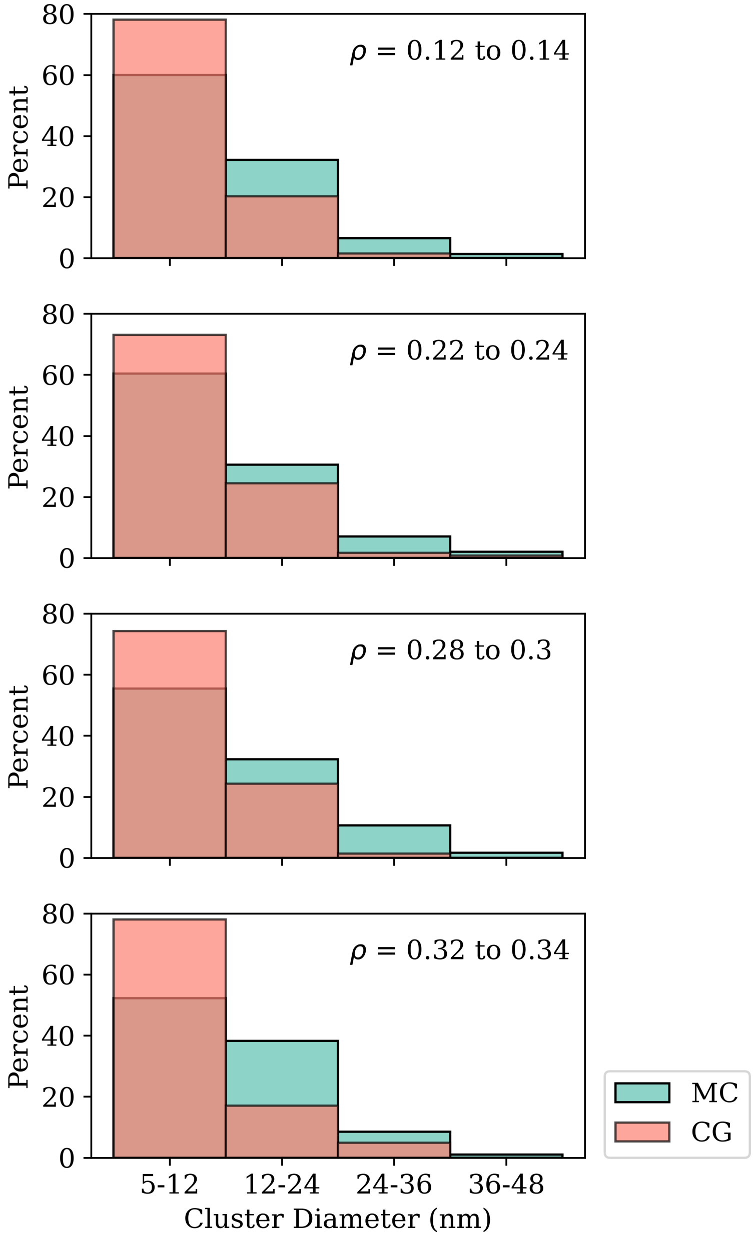

Fig. S8. We compare the cluster size distributions of chromatin configurations derived from our chain growth algorithm (not accounting for nucleosome sterics) with those predicted by MC simulation (adjusting for nucleosome sterics). Plots labeled with “CG” and “MC” correspond to our chain growth algorithm and MC simulations, respectively. The plots include cluster size distributions from models with a -9.66 $k_B T$ HP1 chemical potential and varying H3K9me3 methylation fractions (indicated by ρ). As steric interactions between nucleosomes are accounted for, cluster sizes tend to increase. This results in rightward shifts in the cluster size distributions.

References

1. The ENCODE Project Consortium, An integrated encyclopedia of DNA elements in the human genome. *Nature* **489**, 57–74 (2012).
2. Y Luo, et al., New developments on the encyclopedia of DNA elements (ENCODE) data portal. *Nucleic Acids Res.* **48**, D882–D889 (2020-01-08).
3. BC Hitz, et al., The encode uniform analysis pipelines. *bioRxiv* (2023).
4. Q MacPherson, B Beltran, AJ Spakowitz, Bottom–up modeling of chromatin segregation due to epigenetic modifications. *Proc. Natl. Acad. Sci.* **115**, 12739–12744 (2018-12-11).
5. EF Koslover, AJ Spakowitz, Discretizing elastic chains for coarse-grained polymer models. *Soft Matter* **9**, 7016–7027 (2013).
6. B Beltran, D Kannan, Q MacPherson, AJ Spakowitz, Geometrical heterogeneity dominates thermal fluctuations in facilitating chromatin contacts. *Phys. Rev. Lett.* **123**, 208103 (2019-11-15).
